# New developments during the COVID-19 pandemic: Drama therapy as an online psychological intervention tool

**DOI:** 10.3389/fpsyg.2022.930002

**Published:** 2022-11-10

**Authors:** Zhongyu Shi, Xiuming Jing

**Affiliations:** College of Creative Culture and Communication, Zhejiang Normal University, Jinhua, China

**Keywords:** psychological intervention, creative art therapies, online mode, drama therapy, COVID-19

## Abstract

The COVID-19 pandemic has caused many art therapists and psychotherapists to change their practice modes and workplace setup. As a creative arts therapy (CAT), drama therapy has also shifted to an online mode—a shift that has been highly consequential for practice. This *paper* reviews the existing practice of tele-CAT and tele-drama therapy, explores the advantages and disadvantages of online drama therapy as a psychological intervention tool, and puts forward some developmental suggestions for online drama therapy.

## Introduction

During the COVID-19 pandemic, many art therapists and psychotherapists have had to change their practice modes and workplace setup (Sajnani, 2020). Large-scale isolation measures have led to the adoption of creative art teletherapies (McBeath et al., 2020). Globally, mental health service providers have begun offering remote psychotherapy by telephone or online (Humer et al., 2020; Zubala and Hackett, 2020). This change is significant for promoting and practicing creative art therapies (CATs), which use body consciousness and creative expression to promote individual physical and mental development and thus social welfare (Shafir et al., 2020). CATs cover a range of disciplines, including art therapy, drama therapy, psychodrama, dance/movement therapy, music therapy, and poetry therapy (Jones, 2010; Dieterich-Hartwell and Koch, 2017).

Prominent among the CATs, drama therapy is an active and experiential psychotherapy modality that intentionally and systematically uses drama skills, such as role-play, storytelling, and projective tools, to achieve psychological growth and change (Landy, 1994; Emunah and Johnson, 2009; Feniger-Schaal and Orkibi, 2020; Shafir et al., 2020). It is used to improve anxiety, depression, isolation, social and emotional learning skills, and overall health. Drama therapy is based on the recognition of therapeutic aspects in drama, including creativity, games, exploration, and performance of roles and behaviors (Jones, 1996; Sajnani and Johnson, 2014). Drama therapists use various techniques, such as storytelling, improvisation, masks, ceremonies, and others (Rubenstein, 2005; Weber and Haen, 2005).

Countries have implemented strict public health measures to curb COVID-19 (Adhikari et al., 2020), which has increased psychological pressure and symptoms of mental illness, such as depression and anxiety (Bao et al., 2020; Palgi et al., 2020)—especially among vulnerable groups, such as people with disabilities and victims of domestic violence (Turk et al., 2020; Usher et al., 2020). Epidemics and ensuing mental health crises may be powerful catalysts promoting long-distance psychotherapy.

Research has shown that online psychotherapy can be as effective as face-to-face therapy (Atsmon et al., 2022). It can be more effective for some patients, for instance allowing some patients with autism spectrum disorder (ASD) to participate passively, reducing social interaction pressure (Reyes, 2022). It also reduces therapy-associated costs of transportation, work delays, and childcare (Tuerk et al., 2018; Georgia Salivar et al., 2020). Although tele-psychotherapy has limitations associated with technical issues and impersonality, its prospects are promising.

However, few empirical studies have been conducted on online CATs, a relatively new field of tele-psychotherapy. In existing studies, the following conclusions were drawn. During the COVID-19 epidemic in Massachusetts, investigators used participatory action research (PAR) and inductive topic analysis (ITA) to demonstrate that group drama tele-therapy improved online participants' mental health (Wood et al., 2020). In addition, 1,534 samples were collected by researchers and found that the respondents' perceptions and attitudes toward distance creative arts therapies were different (Feniger-Schaal et al., 2022). A survey in South Africa during COVID-19 showed that people appreciate online art therapy, but had specific concerns regarding challenges (Zubala and Hackett, 2020). A case study found that drama tele-therapy helped older adults to cope with social distancing and experiences of loneliness during *COVID*-19 (Kordova and Keisari, 2020). Based on the analysis of 20 interviews with drama therapy practitioners *from 1*9 countries, Atsmon et al. summed up *four* patient attitudes toward online drama therapy—resistance, anxiety, adaptation, and fluency—and concluded that online drama therapy was a *via*ble branch of drama therapy (Atsmon et al., 2022). Nery's research used online action methods, such as psychodrama, social drama, and spontaneous drama, to stimulate clients' imagination in a virtual environment (Nery, 2022). Reyes conducted a literature review on drama therapy for students with ASD during COVID-19 and found that, although remote technology made some students anxious, it kept them in touch with other people (Reyes, 2022).

This study evaluates online drama therapy as an online psychological intervention tool and provides suggestions for its development.

## Advantages and limitations

### Advantages

Previous researchers have used qualitative or quantitative measurement methods and standardized scales to analyze the emotional performance of online drama therapy clients and therapists. They have identified positive psychological phenomena brought by online drama therapy, as follows.

First, online drama therapy enhances “personal insight,” helping clients freely reveal themselves (Yalom and Leszcz, 2020). Sitting in front of the lens, participants feel they are being gazed at by an audience and pay more attention to aspects that can be captured by webcams, such as facial expressions, rhythm, and intonation (Weinberg, 2020). In a case study, participant Beatrice reported that she began to focus on how to show her image to the camera and was able to contemplate herself from a new angle: “In the case of online psychodrama, you look at yourself … so certainly it is much more intense” (Biancalani et al., 2021, p. 4). Johnson, a practicing psychotherapist, gradually gave the initiative to clients in a drama therapy group composed of *six* patients with schizophrenia, who eventually showed more emotional expressions and felt more intimate and secure than before (Johnson, 1982). These examples show that when individuals become more capable of organizing and adjusting their own behaviors to the environment, the demand for external structures, such as therapists, will gradually decrease. In online drama therapy, which is substantially based on this principle, receiving psychotherapy in a private space already helps create a relatively relaxed, free atmosphere. The therapist can help clients create a safe, emotionally stable environment, enhance their inner strength, and express their emotions.

Moreover, online drama therapy broadens the therapeutic space to living rooms, bedrooms, and other private areas. Its mode brings freshness to psychological feelings, stimulates the desire to perform tasks, and improves the mind's perception of the environment. “We are now showing our most intimate privacy… where we live. The body expands far beyond the skin, which is no longer our only external cover but the whole place is.” (Atsmon et al., 2022, p. 5). In one study, the patient awakened her inner sense of connection by imagining she was introducing the house to the therapist (Kordova and Keisari, 2020).

Online psychological intervention is less affected by life conditions, such as schedules and fixed times and routines, which bring clients a great sense of security, which helps them keep in touch with the group and enhances their sense of belonging (Nery, 2022). Sharing concerns and feelings within the group strengthened group integration during COVID-19 (Carroll et al., 2000) and provided a sense of continuity. Amelia, who has been participating in the group for 5 months, thought the online mode gave her a sense of continuity in the group (Biancalani et al., 2021).

Additionally, online drama therapy requires people to mobilize their imagination and creativity to achieve self-expression and emotional regulation (Armstrong et al., 2019). Online work has inspired many therapists to think more creatively about their knowledge and skills (Wood et al., 2020). For example, they can use digital *photo*graphs to create digital *photograph* collages with older adults (Keisari et al., 2021), perform yoga and warm-up exercises, for example with teenagers in drug abuse centers (Adges, 2020) or with mental health clients (Buckley, 2020), or sing together and exerting creative self-efficacy in a diversified way (Tierney and Farmer, 2002); at the same time, clients' sense of existence is reduced, so that therapists can quantify and evaluate their emotions more clearly as they exert their imagination to make themselves more involved (McBeath et al., 2020).

### Limitations

Online drama therapy is sometimes blocked by objective technical obstacles (Atsmon et al., 2022; Feniger-Schaal et al., 2022), which can reduce clients' motivation for treatment. Many interviewees initially had little confidence in the feasibility, effectiveness, and desirability of online drama therapy (Zubala and Hackett, 2020). Some interviewees think that the Internet connection in online mode is not very good and they have no privacy (Atsmon et al., 2022). At the same time, for other clients, the online mode only increased their anxiety level, and they found it intimidating to communicate through Zoom squares on the screen. They became anxious because they did not know when the therapist would call them (Reyes, 2022).

People find it can be hard to immerse themselves in tele-drama therapy and lack a sense of security when engaged in it because they feel as though their privacy is not guaranteed. When teletherapy is conducted in the client's workplace, the boundaries between the workplace and the client's private space become blurred, and the private information around the client will also be exposed, which may make them feel uneasy (Martinez-Martin et al., 2020). With an increase in interference factors, people probably cannot fully immerse themselves in online drama therapy.

Online drama therapy may intensify people's sense of strangeness and reduce their embodied perception owing to screen barriers. Compared with face-to-face communication, which may alleviate people's oversensitivity and lessen the effects of negative social cues to a certain extent, online drama therapy is limited by the environment and visibility of body language and body cues (Zubala and Hackett, 2020). Consequently, people are less enthusiastic about online modes as they can't really meet (Feniger-Schaal et al., 2022). More specifically, some participants think that warmth between people cannot be felt on the Internet (Biancalani et al., 2021). Certainly, smells and pheromones cannot be transmitted between people through the Internet, which hurts their sense of intimacy and attachment (Cozolino, 2006).

The technical updates brought about by online drama therapy have aggravated some people's sense of psychological exile, especially among older adults. Older adults or other vulnerable groups may encounter difficulties in adapting to new technologies (Kordova and Keisari, 2020). Some therapists are also anxious about connectivity problems and the time lag of the network. When therapists lack confidence in their skills, they have a lower sense of job satisfaction (McBeath et al., 2020).

## Discussion

Judging from the common psychological experiences and emotional representations extracted from quantitative and qualitative data, online drama therapy works as a new media form that has changed people's psychotherapy experiences. However, many people who cannot adapt to advanced techniques feel estranged, feeling that this method lacks a human touch, and hoping to return to the conventional face-to-face form of therapy.

With training programs widely offering digital courses and certifications in online drama therapy (Pilgrim et al., 2020), it has now been promoted and developed as a professional direction for tele-psychotherapy. The continuous practice has evolved into a new paradigm in drama therapy (Johnson and Emunah, 2020). Many therapists expect telecommuting to be part of their core services for clients in the future.

Faced with the popularity of network therapy technology, therapists should modify the experimental paradigm to better adapt to transmission by a screen. The use of writing tools is an example of drama therapy in a virtual environment. Clients and therapists use Google Docs to share creative ideas and write stories (Reyes, 2022). Alternatively, therapists can start from a more personal point of view, such as the client's temperament, affective style, and own advantages and disadvantages, to specifically analyze the client's psychological problems (Landy, 2006). Therapists could also develop more accurate measurement tools to better evaluate clients' emotions and reduce the judgment deviation between online mode and face-to-face mode. For example, Cook conducted quantitative research to explore drama production to stimulate clients' self-promotion skills and self-confidence (Cook, 2020). He created the DTRPI, which is a measurement tool used in research investigation divided into four categories to allow participants to explore role-playing: following directions, concentration, spontaneity, and decisiveness. In the psychotherapy process, the therapists can use video recording, written reports, or other means to ensure the implementation fidelity of the entire psychological intervention (Ang et al., 2018), as *well* as creative art teletherapies. Implementation fidelity refers to the extent to which the core components of intervention measures are implemented according to the plan's intention (Gearing et al., 2011). Therefore, implementation fidelity is necessary to explain the therapeutic effect accurately (Perepletchikova and Kazdin, 2005).

Therapists should actively try the above methods to compensate for people's psychological insecurity caused by technical restrictions and environmental interventions in the online drama therapy process, to encourage clients to trust and support the development of this new technology. As a fairly novel online psychological intervention method, drama therapy, its affordances, and how it can play a better role in the pandemic era are worthy of further research and discussion.

## Author contributions

ZS contributed to the study's conception and design and wrote the manuscript's draft. XJ contributed to the manuscript revision. All authors approved the submitted version.

## Conflict of interest

The authors declare that the research was conducted in the absence of any commercial or financial relationships that could be construed as a potential conflict of interest.

## Publisher's note

All claims expressed in this article are solely those of the authors and do not necessarily represent those of their affiliated organizations, or those of the publisher, the editors and the reviewers. Any product that may be evaluated in this article, or claim that may be made by its manufacturer, is not guaranteed or endorsed by the publisher.
